# Evolution of ligand specificity in vertebrate corticosteroid receptors

**DOI:** 10.1186/1471-2148-11-14

**Published:** 2011-01-14

**Authors:** Adam S Arterbery, Daniel J Fergus, Elizabeth A Fogarty, John Mayberry, David L Deitcher, W Lee Kraus, Andrew H Bass

**Affiliations:** 1 Department of Neurobiology and Behavior, Cornell University, Ithaca, NY, 14853, USA; 2 Department of Molecular Biology and Genetics, Cornell University, Ithaca, NY, 14853, USA; 3 Department of Mathematics, University of the Pacific, Stockton, CA, 95211, USA

## Abstract

**Background:**

Corticosteroid receptors include mineralocorticoid (MR) and glucocorticoid (GR) receptors. Teleost fishes have a single MR and duplicate GRs that show variable sensitivities to mineralocorticoids and glucocorticoids. How these receptors compare functionally to tetrapod MR and GR, and the evolutionary significance of maintaining two GRs, remains unclear.

**Results:**

We used up to seven steroids (including aldosterone, cortisol and 11-deoxycorticosterone [DOC]) to compare the ligand specificity of the ligand binding domains of corticosteroid receptors between a mammal (*Mus musculus*) and the midshipman fish (*Porichthys notatus*), a teleost model for steroid regulation of neural and behavioral plasticity. Variation in mineralocorticoid sensitivity was considered in a broader phylogenetic context by examining the aldosterone sensitivity of MR and GRs from the distantly related daffodil cichlid (*Neolamprologus pulcher*), another teleost model for neurobehavioral plasticity. Both teleost species had a single MR and duplicate GRs. All MRs were sensitive to DOC, consistent with the hypothesis that DOC was the initial ligand of the ancestral MR. Variation in GR steroid-specificity corresponds to nine identified amino acid residue substitutions rather than phylogenetic relationships based on receptor sequences.

**Conclusion:**

The mineralocorticoid sensitivity of duplicate GRs in teleosts is highly labile in the context of their evolutionary phylogeny, a property that likely led to neo-functionalization and maintenance of two GRs.

## Background

The nuclear receptor super-family of proteins includes steroid receptors that are ligand-activated transcription factors mediating steroid actions on a multitude of behavioral, morphological and physiological processes [1]. The two major functional groups of vertebrate corticosteroid receptors are glucocorticoid (GR) and mineralocorticoid (MR) receptors distinguished by their amino acid sequences and ligand specificity. Jawless vertebrates (hagfish and lamprey) have a single corticosteroid receptor, while jawed cartilaginous and bony fishes possess GRs and MRs [2,3]. Bony fishes fall into two main clades: lobe-finned that includes tetrapods and ray-finned that includes teleosts, the most species-rich group of vertebrates [4]. Functional relationships between the GRs and MRs of tetrapods and teleosts remain somewhat uncertain due, in part, to a lack of consensus as to the bioactive mineralocorticoid ligand and the presence of two GRs in some teleosts. Here, we compare the ligand specificity of GR and MR ligand binding domains (LBD) between mammals and teleosts, presenting new evidence to support the hypothesis that neo-functionalization likely facilitated the maintenance of duplicate GRs in teleosts following an initial duplication event with the origin of bony fishes. Focusing on the LBD allowed us to compare variation in ligand binding without the confounding effects of variation in other domains, such as the DNA binding domain (DBD) and N-terminal region that can alter transactivational activity [5].

Among most mammals, including humans, cortisol is the principal glucocorticoid that induces the transcriptional activities of both the GR and MR [1]. The mineralocorticoid aldosterone also activates MR, but not GR [1,6]. Like mammals, cortisol is the major glucocorticoid among teleosts [1]. However, unlike mammals, there is no convincing evidence for significant circulating levels of aldosterone among teleosts [7-9]; the aldosterone precursor 11-deoxycorticosterone (DOC) is a prominent candidate for the dominant mineralocorticoid in teleosts [10]. Most teleosts have two GRs [11-13], with some being cortisol-specific and others activated *in vitro *by both aldosterone and cortisol in some species [12]. The existence of teleost GRs with aldosterone sensitivity, but where cortisol may be the predominant ligand in the absence of aldosterone, has prompted investigations like the current one into the functional and evolutionary relationships of corticosteroid receptors and the ligands that induce their transcriptional activation.

To rigorously investigate the functional diversity of corticosteroid receptors, we compared the dose-dependent ligand sensitivity of the LBD of MRs and GRs between a teleost, the midshipman (*Porichthys notatus*) [4], and a mammal, the mouse (*Mus musculus*). Midshipman fish are a key model for steroid modulation of reproductive behavior at multiple levels of analysis ranging from behavioral and molecular neuroendocrinology to neurophysiology [14,15]. Inclusion of the midshipman, from which a partial GR (GR1) and partial MR sequences have been published [16], and for which we report a second GR (GR2) here, also provided us the opportunity to compare ligand specificities of MRs and GRs between distantly related teleosts using a single assay. To this end, we examined aldosterone and cortisol activation of MR, GR1, and GR2 from a cichlid (*Neolamprologous pulcher*). Cichlids are a representative of the largest order of teleosts (Perciformes) [4], have duplicate GRs [12], and are another teleost model for the behavioral neuroendocrinology of reproductive plasticity [17-19].

Since multiple domains of steroid receptor gene products can influence transcriptional activity [20-23], we cloned the LBD of each receptor in frame with the DNA binding domain (DBD) of the yeast transcription factor GAL4. We employed a GAL4-UAS promoter to control expression of a luciferase reporter gene, similar to previous studies in heterologous cell systems [2,24,25], as well as *in vivo *[26]. This system allowed us to focus on variation in ligand binding independent of variation in other properties of the receptor [See: [27]]. Using a battery of up to seven steroids, we show differences in the LBD specificity of duplicate teleost GRs, as well as the GRs between teleosts and mammals. When viewed broadly in the context of a phylogenetic analysis of corticosteroid receptors, the results suggest that duplicate GRs among teleosts exhibit an evolutionarily labile pattern of ligand binding that is unique among the major vertebrate lineages with one glucocorticoid-specific GR and another GR with more general corticosteroid sensitivity, similar to the ancestral vertebrate MR.

## Results

### Phylogenetic analysis of corticosteroid receptors

To more completely characterize the evolutionary relationships between the corticosteroid receptors of our main study species and other vertebrates, we first cloned and sequenced the full LBD with the hinge region and a short portion of the DBDs of midshipman MR (GenBank no. GU384923.1) and GR2 (GenBank no. HM164445). The resulting sequences were aligned with multiple corticosteroid receptors to verify the identity and similarity of the cloned genes with their homologs from other species. The cloned regions of the corticosteroid receptors included the entire coding region for the LBD, hinge region, and a few amino acids of the DBD sequence.

**Figure 1:**
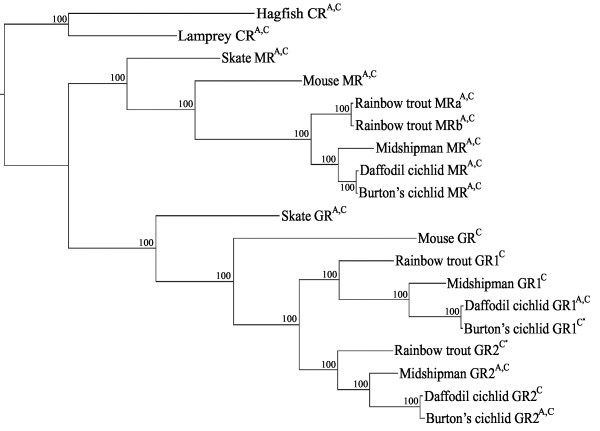
**Phylogram of the evolutionary relationship of corticosteroid receptors based on DNA sequence identity**. In addition to the receptors examined in this study (mouse, *Mus musculus*; midshipman fish, *Porichthys notatus*; daffodil cichlid, *Neolamprologous pulcher*), we included GRs and MRs from another cichlid (Burton's cichlid, *Astatotilapia burtoni*); a cartilaginous fish, the little skate (*Leucoraja erinacea*); and rainbow trout (*Oncorhynchus mykiss*) which have all been used in similar transactivational studies [2,12,13,25]. The Burton's cichlid GRs are named according to a revised nomenclature [29]. Also included are the general corticosteroid receptor of a hagfish (*Myxine glutinosa*) and lamprey (*Petromyzon marinus*) that are evolutionarily basal to the radiation of GRs and MRs. The superscripts 'A' and 'C' indicate sensitivity to aldosterone and cortisol at ≤ 10^-7 ^M based on our results and previous studies. *Aldosterone sensitivities of Burton's cichlid GR1 and rainbow trout GR2 are somewhat unclear [2,12,13].

A phylogram of corticosteroid receptor sequences had 100% posterior probabilities at each node (Figure 1). Due to a discrepancy between the names and the phylogenetic relationship of Burton's cichlid GR1 and GR2 relative to other teleosts [11,28], a recent report renamed their GRs [29]. We utilized this more recent and phylogenetically consistent naming system. As suggested previously [2,11,28,30], the results indicated that the ancestral corticosteroid receptor (represented by hagfish and lamprey CR) underwent a duplication event giving rise to GR and MR prior to the divergence of cartilaginous (represented here by skate) and bony fishes that includes sarcopterygians/lobe-finned fish that gave rise to tetrapods (represented here by mouse). A second duplication event resulted in GR1 and GR2 within the second major clade of bony fish, the actinopterygian/ray-finned fishes (represented by trout, cichlids and midshipman).

### Ligand specificity

**Figure 2:**
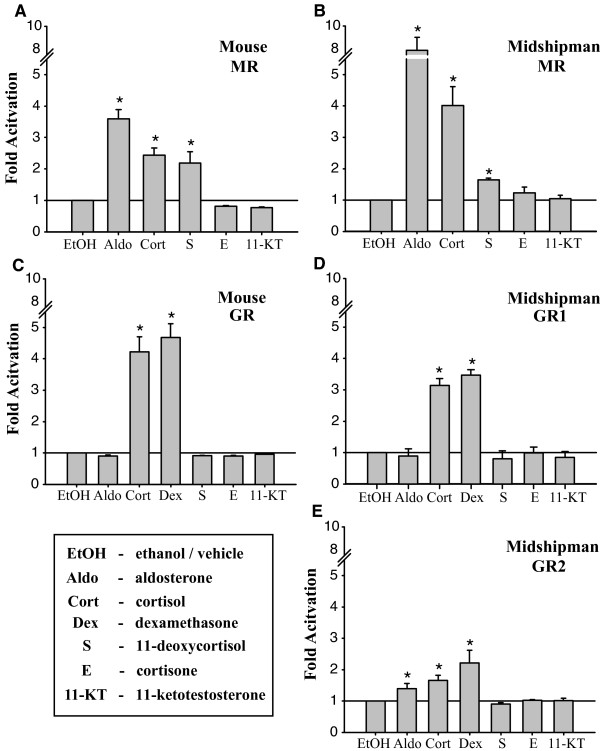
**Corticosteroid receptor ligand specificity**. Graphs showing relative fold-activation over ethanol for midshipman and mouse LBD constructs to 10^-7 ^M of each steroid treatment. MRs (A, B) showed expected sensitivity to both cortisol and aldosterone. GRs (C-E) were all sensitive to cortisol, but showed variable sensitivity to aldosterone. None of the receptors were activated by either cortisone or 11-ketotestosterone (11-KT).

Using a heterologous expression system, we tested the activity of corticosteroid receptor homologues induced by a variety of steroid hormones found among vertebrates. To do this we first confirmed expression of the full-length LBD-GAL4 fusion products from the plasmid constructs by Western blotting, which showed bands of the expected sizes for each of the constructs (data not shown). As an initial screen for ligand specificity and efficacy of the expression system, we measured the luciferase activity produced by cells co-transfected with each of the LBD-GAL4 constructs and the luciferase reporter construct in response to hormone treatments at one concentration, 10^-7 ^M. Normalized values were expressed as fold change over vehicle (ethanol) treatment (Figure 2). Significant ligand induced activities were observed for all the receptor LBDs examined. The MR constructs from both midshipman and mouse showed significant activation in response to 10^-7 ^M concentrations of aldosterone and cortisol (Figures 2A, and 2B). The cortisol precursor 11-deoxycortisol (Reichstein's compound S) also produced activity significantly above baseline (Figures 2A and 2B). Mouse GR LBD was sensitive only to cortisol and the synthetic glucocorticoid dexamethasone but not to the mineralocorticoid aldosterone (Figure 2C), consistent with previous results from rats [2]. Midshipman GR1 (Figure 2D) behaved similar to mouse GR, with significant activity induced only by cortisol and dexamethasone. Midshipman GR2 was also activated by cortisol and dexamethasone, but like mouse and midshipman MRs showed significant activity in response to 10^-7 ^M of aldosterone (Figure 2E).

**Figure 3:**
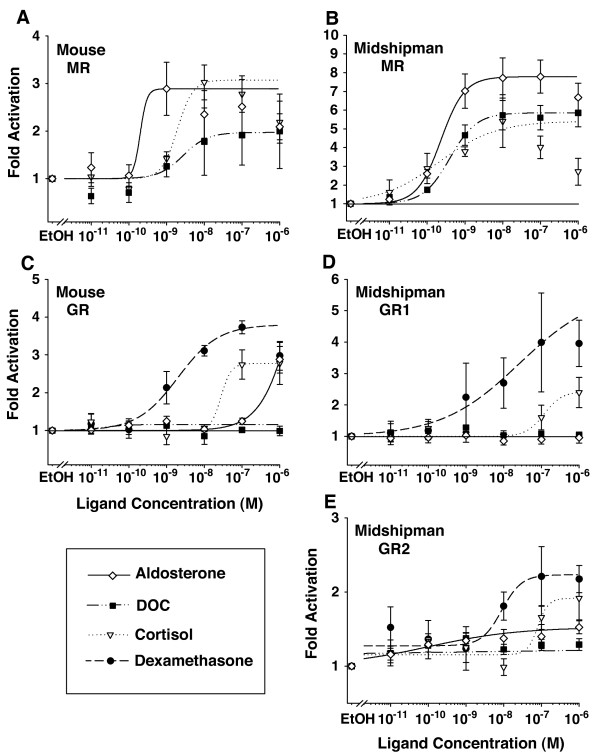
**Dose response curves of corticosteroid receptors**. (A-E) Dose response curves characterized the sensitivity of mouse and midshipman MRs and GRs for aldosterone, 11-deoxycorticosterone (DOC), cortisol, and dexamethasone (see legend, lower left). The MR LBD constructs of both (A) mouse and (B) midshipman were sensitive to DOC, a precursor in the aldosterone biosynthetic pathway. (C) The mouse GR LBD was activated by cortisol, dexamethasone, and very high doses of aldosterone (> 10^-7 ^M), yet did not show a response to DOC. (D) Midshipman GR1 LBD showed very strong glucocorticoid specificity, while (E) the GR2 LBD showed low activation by all the corticosteroids at even very low doses, with increased activity at higher doses of dexamethasone and cortisol.

The results of this initial screening demonstrated that: (1) the LBDs were sufficient to confer ligand specificity, (2) the GRs showed strong specificity for cortisol over its upstream and downstream biosynthetic products 11-deoxycortisol and cortisone (Reichstein's compound E, Figure 2), and (3) aldosterone sensitivity was variable among the GRs, found only for GR2.

### Ligand sensitivity

To rigorously characterize the ligand sensitivity of the receptors, we performed dose response experiments with the LBD constructs from the MRs and GRs of the mouse and midshipman using cortisol, DOC and aldosterone as well as dexamethasone for the GRs (Figures 3). As noted earlier, DOC was included because it has been suggested that this steroid, a precursor to aldosterone in tetrapods, may function as an important agonist of MRs in teleost fish that lack aldosterone [1,2,10].

We estimated EC_50 _values (see Table 1) from the logistic dose response curves shown in Figure 3. For several of the responses here, such as midshipman MR with cortisol, receptor activation initially increased and then decreased with increasing ligand doses, a trend previously observed in corticosteroid receptors [e.g., [12,31]]. This may be indicative of cytotoxicity at high steroid doses [32]. This reduced activity at higher doses leads to underestimated upper bounds in the fitted logistic curves that, in turn, yield underestimates of the corresponding EC_50 _values. To counter this effect when performing our logistic fits, we excluded doses at which the observed activity decreased relative to lower doses (Table 1, Figure 3).

**Table 1:** Corticosteroid receptor EC_50 _values

	Aldosterone	DOC	Cortisol	Dexamethasone
Mouse MR	0.20	2.75	1.94	

Midshipman MR	0.23	0.40	0.20	

Mouse GR	>1000	--	27.7	2.00

Midshipman GR1	--	--	118.5	5.76

Midshipman GR2	ND	ND	85.9	8.95

Note: EC_50 _ligand nanomolar concentrations based on logistic regressions. No response is indicated by: --. ND indicates that the EC_50 _could not be determined based on the curve.

As in the initial screen, MR LBDs were sensitive to both aldosterone and cortisol (Figures 3A and 3B). The EC_50 _of cortisol was an order of magnitude higher than that of aldosterone for the mouse MR, while the EC_50_s for aldosterone and cortisol were approximately the same as for midshipman MR (Table 1). However, the maximum fold activation induced by aldosterone was nearly twice that induced by cortisol for midshipman MR (Figures 3A and 3B). Therefore, while the EC_50_s of cortisol and aldosterone did not differ substantially, the level of activity induced at the EC_50 _was much higher with aldosterone. DOC also induced activity of both mouse and midshipman MR constructs. The dose response curve for DOC in midshipman was very similar to that of cortisol (Figure 3B). DOC did not activate mouse MR to the same maximal level as either aldosterone or cortisol and the EC_50 _of DOC, like that of cortisol, was approximately an order of magnitude higher than that of aldosterone.

The GR LBD constructs were most sensitive to dexamethasone (Figures 3C, D and 3E; Table 1), as reported previously for full length GRs [11,13,33]. This high dexamethasone sensitivity was evident in both the maximum observed activation as well as the EC_50 _values. For all GRs, the EC_50 _for cortisol was approximately an order of magnitude, or more, greater than that of dexamethasone. The response to cortisol was fairly similar across GRs, with a somewhat lower EC_50 _for mouse GR than either midshipman GRs.

The largest distinction between the GR constructs was in the activation by aldosterone and DOC. While mouse GR was not activated by aldosterone in our initial screen using 10^­7 ^M aldosterone (Figure 2A), 10^­6 ^M aldosterone caused substantial activation (Figure 3C) as seen previously for rat GR [2]. DOC, however, failed to activate this receptor at any concentration. The midshipman GR1 construct showed no response to either aldosterone or DOC at any of the doses examined (Figure 3D). In contrast to midshipman GR1, midshipman GR2 was sensitive to both aldosterone and DOC (Figure 3E). Neither of these ligands induced the levels of response in GR2 that we observed with either dexamethasone or cortisol. The sensitivity of the GR2 construct to low doses of aldosterone and DOC made accurate EC_50 _estimates impossible (Figure 3E, Table 1). None of the LBD constructs were activated by either cortisone or 11-KT (Figure 2), indicating that the low sensitivity of the GR2 construct to aldosterone and DOC did not reflect this artificial construct's overall lack of specificity. Rather, this likely reflected true mineralocorticoid sensitivity of midshipman GR2.

GAL4-LBD assays, like those used here and in other studies [2,25,34], allowed us to assess variation in LBD activation, while eliminating differences in activity that may result from variation in the N-terminal A/B domains or DBDs [5]. Though the remainder of the receptor may modify ligand responses, our results using GAL4-LBD constructs likely reflected the activity of the full-length receptor (e.g., the sensitivity of the midshipman MR was similar to that for full-length MRs of other teleosts [10,12]).

### Amino acid substitutions and GR ligand specificity

**Figure 4:**
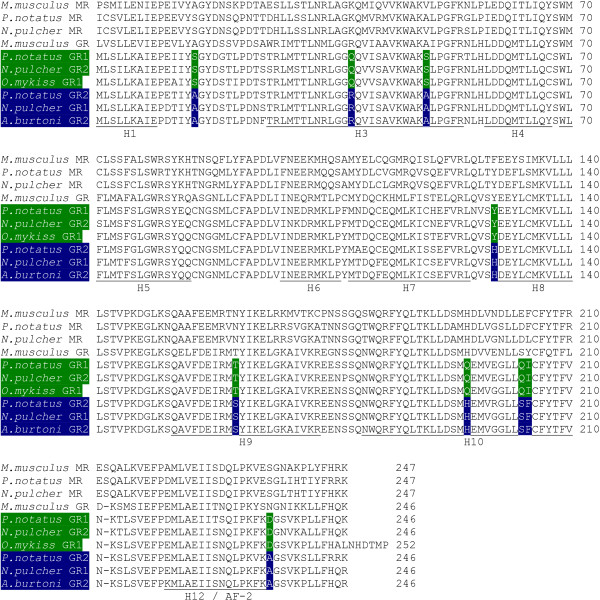
**LBD alignment**. The alignment of the LBDs of the MRs and GRs used in this study demonstrates the high sequence identity between genes. Sequence locations of the secondary structure helices are given below the alignment. To identify amino acids that may play a role in mineralocorticoid sensitivity of the duplicate teleost GRs, we aligned the LBDs of GRs that are mineralocorticoid insensitive (green) and GRs that show mineralocorticoid sensitivity (blue). We identified nine positions (highlighted within the alignment) with amino acid residues conserved within but not between aldosterone-sensitive and insensitive groups. These residues included positions 15, 38, 49, 129, 161, 195, 203, 204 and 235. (Position 1 of the LBDs corresponds to positions 538 and 532 of the full-length mouse and human GRs, respectively.)

Activation of the duplicate GRs by DOC and/or aldosterone was not consistent with the phylogenetic relationships of the receptors (Figure 1, Table 2). For example, the aldosterone sensitivity of GR1 and of GR2 varied between cichlid species (Figure 1). This suggested that mineralocorticoid sensitivity was evolutionarily plastic and may have resulted from a small number of amino acid substitutions in the GRs rather than overall sequence homology. Strong candidates for amino acids regulating mineralocorticoid sensitivity are those that are conserved between GRs with similar mineralocorticoid sensitivities, but not between GRs with differing sensitivities, independent of phylogeny. We compared the mineralocorticoid-sensitive GR LBD sequences from Burton's cichlid GR2 and midshipman GR2 with the mineralocorticoid-insensitive rainbow trout GR1 and midshipman GR1 (mineralocorticoid insensitive and sensitive indicated by green and blue, respectively, in Figure 4). Nine amino acids were conserved within, but not between, these groups (A/S15, R/Q38, A/S49, H/Y129, S/T161, H/Q195, S/Q203, F/I204 and A/D235; Figure 4).

**Table 2:** Corticosteroid receptor ligand specificity

	Aldo	DOC	Cortisol	Dex	S	E
Hagfish CR [2]	+	+	+	N.A.	+	N.A.

Lamprey CR [2]	+	+	+	N.A.	+	N.A.

Skate MR [2,25]	+	+	+		+	N.A.

Mouse MR	+	+	+		+	0

Rainbow Trout MR [10]	+	+	+		+	N.A.

Burton's cichlid MR [2,12]	+	N.A.	+		N.A.	N.A.

Daffodil cichlid MR	+	N.A.	+		N.A.	N.A.

Midshipman MR	+	+	+		+	0

Skate GR [2,25]	+	+	+	N.A.	+	

Mouse GR	0 ^1^	0	+	+	0	0

Rainbow trout GR1 [13]	0	0	+	+	0	0

Burton's cichlid GR1 [2,12]	+/0 ^2^	N.A.	+	N.A.	N.A.	N.A.

Daffodil cichlid GR1	+	N.A.	+	N.A.	N.A.	N.A.

Midshipman GR1	0	0	+	+	0	0

Rainbow trout GR2 [13]	0 ^3^	0	+	+	+	0

Burton's cichlid GR2 [12]	+	N.A.	+	N.A.	N.A.	N.A.

Daffodil cichlid GR2	0	N.A.	+	N.A.	N.A.	N.A.

Midshipman GR2	+	+	+	+	0	0

Note: Aldo, aldosterone; DOC, 11-deoxycorticosterone; Dex, dexamethasone; Reichstein's compound S, 11-deoxycortisol; Reichstein's compound E, cortisone. + and 0 indicate activation or lack of activation, respectively, by each ligand to induce transactivational activity at concentrations of ≤ 10^-7 ^M. N.A. indicates that the data are not available. Data from previous studies are indicated by the citations following the receptor type. The MR response to dexamethasone is excluded because of the confounding dexamethasone paradox [53].

^1^Activity at >10^-7 ^M;

^2^Disagreement between previous studies;

^3^Possible low activation at 10^-6 ^M

**Figure 5:**
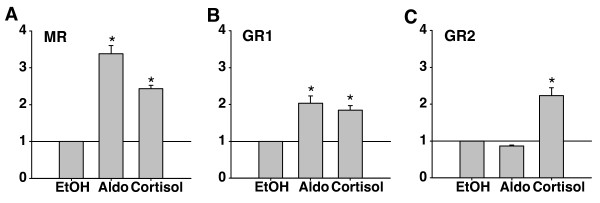
**Specificity of Daffodil cichlid corticosteroid receptors**. Graphs showing relative fold-activation over ethanol for daffodil cichlid (*Neolamprologous pulcher*) LBD constructs to 10^-7 ^M of ethanol [EtOH], aldosterone [Aldo], and cortisol. MR (A) showed expected sensitivity to cortisol and aldosterone. GR1 (B) was also activated by both aldosterone and cortisol, while GR2 (C) was specific for cortisol, with no aldosterone sensitivity. This pattern of GR aldosterone sensitivity is the reverse, phylogenetically, of what was seen for midshipman GRs (Figures 1, 2 and 3).

To test the role of the nine candidate amino acids on GR mineralocorticoid sensitivity, we performed transactivation assays using GAL4 constructs of daffodil cichlid MR, GR1, and GR2 with 10^-7 ^M aldosterone and cortisol (Figure 5; same concentration used in our initial screen, Figure 2). At the nine amino acid positions identified above, the LBD of daffodil cichlid GR1 was found to be identical to midshipman GR2 while daffodil cichlid GR2 was identical to midshipman GR1. The daffodil cichlid MR construct was activated by both aldosterone and cortisol, consistent with midshipman MR and a previous study of full-length Burton's cichlid MR [12]. The daffodil cichlid GR1 construct was activated by both aldosterone and cortisol, while daffodil cichlid GR2 was cortisol-specific at 10^-7^M, consistent with a role of the identified amino acid substitutions in regulating mineralocorticoid sensitivity.

**Figure 6:**
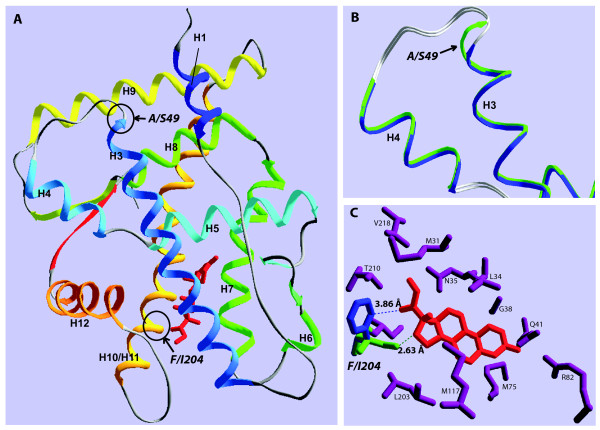
**Protein structure of GR LBDs**. Protein structure homology models were created for midshipman GR1 and GR2 LBDs and indicated two substitutions (A/S49 and F/I204) of particular interest. (A) The overall homology model of the GR1 LBD with bound cortisol (red) indicating the position of these two substitutions. Helices are numbered H1 through H12. (B) The substitution at position 49 lengthens helix 3 in GR1 (green) relative to GR2 (blue) as well as changes the structure at this location. (C) Position 204 is found in the ligand-binding pocket, within 4 Å of the putative bound ligand. The change between isoleucine (GR1, green) and phenylalanine (GR2, blue) represents a large change to the shape of the ligand-binding pocket. Model distances between phenylalanine or isoleucine and the ligand (cortisol, red) are indicated.

We next used protein structure homology modeling to examine the locations of the identified amino acid substitutions and access the likelihood that such substitutions might alter ligand specificity. Modeling the midshipman GR LBDs was based on those of a putative ancestral GR and a general corticosteroid receptor. Two of the nine identified candidate amino acids, A/S49 and F/I204, appeared to be particularly strong candidates for regulating LBD function due to estimated proximity to the bound corticosteroid or to structural change induced in the protein models (Figure 6A). The A/S49 substitution was located at the end of helix 3, distant from the ligand-binding pocket; however, this substitution between polar and non-polar residues altered the length of helix 3 and the loop between helices 3 and 4 (Figure 6B). This may have an important effect on the relative positioning of helix 3 and thus how well the ligand-binding pocket can accommodate either aldosterone or DOC. Furthermore, the side chains of the residues of substitution F/I204 are located less than 4 Å from the putative bound ligand position (Figure 6C). This substitution represents a major change in the physical structure of the ligand-binding pocket of the LBD, as well as a change in the degree of hydrophobicity. Such a change could alter the positioning and the binding of a ligand within the ligand-binding pocket. The seven remaining amino acid substitutions were more distant from the ligand-binding pocket and failed to induce structural changes between the homology models.

## Discussion

We took a broad comparative approach using chimeric GAL4-LBD constructs in a single assay to examine the ligand specificity of the LBDs of the full range of corticosteroid receptors within single species as well as across deeply diverged vertebrate taxa. As discussed below, this property likely led to neo-functionalization and maintenance of two GRs. Comparisons of the amino acid sequences and predicted structures of the duplicate GR LBDs added insight into the evolutionary changes that may regulate ligand specificity. In particular, the mineralocorticoid sensitivities of duplicate GRs in teleosts were highly labile in the context of their molecular phylogeny, i.e., it could not be predicted based on the degree of similarity between their sequences (Figure 1).

### Ligand specificity

Aldosterone is the primary mineralocorticoid in tetrapods [1], while teleost fish appear to lack the aldosterone synthase necessary to produce aldosterone [7]. We demonstrated that teleost MR LBD is sensitive to both mineralocorticoids and glucocorticoids, consistent with previous results for both the LBD and the whole protein [2,10,12]. The maximal activation by aldosterone was greater than that of either DOC or cortisol (Figure 3B), though the variation in EC_50 _values of these ligands for the midshipman MR construct were not substantial (Table 1). DOC, the potential natural agonist for MR in teleosts [10], elicited a response from midshipman MR. This was consistent with results for the full-length MR of rainbow trout [10], the MR LBD of skate [25], and the corticosteroid receptors of the more basal hagfish and lamprey (Table 2, also see Figure 1) [2]. Our data thus support the hypothesis that the evolution of an MR predates aldosterone [2,35].

DOC, which elicits activity from MRs across a range of vertebrates (Table 2), may be the primary ligand of the ancestral MR [8]. Since DOC activates the corticosteroid receptors of jawless vertebrates [2], the proposed ancestral corticosteroid receptor (see Figure 1) [2], the maintained DOC sensitivity of MR may represent a conserved ancestral function. Aldosterone induced greater maximal activation than DOC for both mouse and midshipman MR LBD constructs with lower EC_50_s, suggesting that aldosterone evolved as a more potent MR agonist among lobe-finned fishes, as evidenced in extant lungfish and descendant tetrapods [1]. The response of the mouse MR to DOC (Figure 3A), together with evidence for DOC in the plasma of mammals [e.g., [36,37]], suggests that DOC may still be an important ligand of the tetrapod MR. The MR LBDs studied here showed sensitivity to both aldosterone and cortisol, as well as two steroids involved in the biosynthesis of aldosterone and cortisol (DOC, 11-deoxycortisol) [see: [38]]. This reflects both the overall promiscuity of the MR and conserved sensitivity of the ancestral corticosteroid receptor (Table 2). Three of these steroids (DOC, 11-deoxycortisol, and cortisol) are present in the plasma of teleosts [39]; thus, teleost MR has either a wide array of endogenous ligands or alternate mechanisms inhibit certain steroids from binding to MR, as occurs in mammals [40,41].

### Neo-functionalization of duplicate GRs

The relative ligand sensitivities of GR1 and GR2 in teleosts provide insight into the biological basis for maintaining duplicate GRs. Bury and Sturm [38] hypothesized that the difference in sensitivity between rainbow trout GRs evolved such that GR2 is active at basal cortisol levels while GR1 is recruited under stressful conditions with elevated cortisol. The GR LBDs examined in this study were all activated by cortisol and with a slightly lower EC_50 _for midshipman GR2 than GR1 (Table 1), though not on the order of the difference observed in rainbow trout [13]. The dose-response data showed activity from GR2 at very low cortisol concentrations, while GR1 was not activated at these concentrations (Figures 3D and 3E). This is further consistent with the hypothesis of GR1 activity restricted to times of high cortisol release.

Bury and Sturm [38] described the observed difference in cortisol sensitivity between duplicate GRs as a neo-functionalization and proposed that the functions of a single GR are partitioned between the duplicate GRs into stressful (high cortisol) and basal-level (low cortisol) activity. Such partitioning seems more accurately described as sub-functionalization since neither of the duplicate GRs acquire a novel function. The evidence presented here supports neo-functionalization based on the promiscuity of ligand specificity among GR paralogues and suggests that promiscuous GRs acquired a novel function distinct from their direct ancestral predecessor. As we report for midshipman and daffodil cichlid, it appears that in many teleost species one GR shows strong ligand specificity for glucocorticoids (cortisol and dexamethasone), while the other GR responds to glucocorticoids as well as either aldosterone and DOC or 11-deoxycortisol (Table 2). The broader ligand sensitivity is more similar to that of the single skate GR as well as the basal hagfish and lamprey corticosteroid receptors (Table 2, also see Figure 1). However, because the inferred ancestral GR of bony vertebrates is cortisol-specific [42], the observed promiscuousness among GRs represents a novel property and thus is a neo-functionalization, rather than sub-functionalization of the ancestral characters. A similar reversion of GR to a more broadly sensitive receptor is seen in chickens (*Gallus gallus*) in which the full-length GR is more sensitive to aldosterone than to cortisol [43]. A promiscuous GR among bony vertebrates could function in a role similar to the ancestral GR of cartilaginous fishes (see skate, Figure 1), while a cortisol-specific GR serves a more selective function.

The fact that GR steroid-specificity is not predictable based on the phylogeny of receptor sequences (Figure 1) suggests that activation by steroids other than cortisol is evolutionarily labile, providing a basis for rapid adaptation of these receptors. The evolutionary plasticity of GR specificity may occasionally lead to the duplicate teleost GRs reverting to redundant states and thus one of the receptors being lost in some lineages, which may be responsible for the single GR of zebrafish (*Danio rerio*) [33,44].

### Amino acid substitutions and GR ligand specificity

We aligned the amino acid sequences of LBDs from GR1 and GR2 of midshipman with GR2 from Burton's cichlid and GR1 from rainbow trout to examine the molecular basis of variation in the mineralocorticoid sensitivity of GRs. Nine amino acid positions correlated with mineralocorticoid sensitivity (Figure 4). These positions showed the same correlation with mineralocorticoid sensitivity in the daffodil cichlid GR LBDs (Figures 4 and 5), strongly supporting a role for these residues in altering ligand specificity. Five of the identified residues (R48, A49, H195, S203 and F204) are noteworthy because the same amino acids occur at these positions in the mineralocorticoid-sensitive chicken GR [43], with residues H195 and F204 common in many MRs (Figure 4). Furthermore, residue F204 has previously been shown to directly interact with the ligand, is adjacent to another ligand contacting residue [21,45], and is important for proper receptor co-activator and co-repressor binding [46]. Using homology model structures to midshipman GR1 and GR2, we identified two of the nine amino acid residues as particularly strong candidates for regulating ligand specificity because they likely either make contact with the ligand or change the structure of the LBD (Figure 6). The A/S substitution at position 49 is distant from the ligand-binding pocket, but it alters the length and polarity of the distal end of helix 3 (Figure 6B) which is likely to affect the conformation and flexibility of helix 3 and the loop between helices 3 and 4. Such conformational effects can play an important role in the ligand specificity of corticosteroid receptors [42,47]. Additionally, the F/I substitution at position 204 is located in the binding pocket, within 4Å of the putative ligand position (Figure 6C). Though this is a substitution between two non-polar residues, this substitution introduces a change in the degree of hydrophobicity and a large difference in structure. Such amino acid substitutions within the LBD affect the binding specificity and transactivational response of steroid receptors to particular ligands [21,45].

Our results are consistent with the findings of Bridgham et al [24] that accumulation of restrictive mutations make a reversal of protein structure and function unlikely. None of the nine candidate amino acid residues identified in our analysis correspond to residues previously suggested to play a role in the evolution of GR cortisol specificity [24,42,47]. This incongruity indicated that mineralocorticoid sensitivity among teleost GRs likely results from novel mutations that permit aldosterone binding to otherwise cortisol specific receptors rather than back-mutations to the ancestral aldosterone sensitive state.

## Conclusion

A luciferase reporter assay was used to characterize the transcriptional activation of corticosteroid receptors across the two most widely divergent groups of bony vertebrates, tetrapods and teleosts, showing the activation of all teleost receptors by cortisol with variable activation by other steroids in the corticosteroid biosynthetic pathways. Phylogenetic analyses demonstrated that GR aldosterone-sensitivity cannot be predicted among fishes on the basis of receptor sequence similarity. This suggests that different lines of teleosts have adapted duplicate GRs for divergent functions. While one has cortisol-specificity, the other is broadly sensitive to corticosteroids, more akin to the ancestral state. However, the latter is not a reversal of phenotype, but instead represents a novel condition brought about by specific amino acid substitutions. Such neo-functionalization reflects the dynamic and complex regulation inherent in the divergence of corticosteroid receptors across vertebrates.

## Methods

### Tissue sources

Midshipman were hand collected from sites in northern California during the breeding season (May-August), shipped to Cornell University within 72 h, and maintained in saltwater aquaria until sacrificed within 24-48 h of receipt. Tissue sampling was carried out following deep anesthetization (0.025% benzocaine; Sigma, St. Louis, MO). Mouse liver (*Mus musculus*) was donated by David McCobb (Cornell University) and daffodil cichlid liver (*Neolamprologus pulcher*) by Sigal Balshine (McMaster University). All tissue samples were flash frozen and stored at -80°C until used for RNA extraction. Animal protocols were approved by the Cornell University Institutional Animal Care and Use Committee.

### Cloning of LBDs

RNA was isolated from the tissue samples using Trizol (Invitrogen, Carlsbad, CA) and reverse transcribed using Superscript III Reverse Transcriptase (Invitrogen, Carlsbad, CA) following the manufacturer's protocols. PCR on cDNA from each tissue was conducted using gene/species-specific primers that targeted the LBD of the previously reported corticosteroid receptors: *M. musculus *(mouse) MR (GenBank no. BC133713.1) and GR (GenBank no. X04435.1); *N. pulcher *(daffodil cichlid) MR (GenBank no. EF661650.1), GR1 (GenBank no. EF661652.1), and GR2 (GenBank no. EF661651.1); and *P. notatus *(plainfin midshipman) GR1 (GenBank no. EF092836.2). To acquire the full LBD of the MR and a second GR from midshipman we used degenerate PCR primers designed based on the amino acid sequences of regions that are highly conserved in other species, adding restriction enzyme sites to the 5' end of each primer to aid in cloning. The degenerate MR forward and reverse primer sequences were, respectively: TCAC**GGATCC**GGNTGYCAYTAYGGNGTNGT and CGAT**GAATTC**TYAYTTYYTRTGRAARTA. The initial degenerate GR forward and reverse primers were: TCAC**GGATCC**CARCAYAAYTAYYTNTGYGC and ACT**CCCGGG**TYAYAAYTGRTGRAANARNA. Subsequent to acquiring a partial sequence of midshipman GR2, the following forward primer was used for amplifying and cloning of the full GR2 LBD: AGA**GGATCC**CCAGCCTGCCGCTATCGC. For each primer the regions in bold represent restriction sites used for cloning. PCR amplification was performed using the FailSafe polymerase mix (Epicentre, Madison, WI). Products were ligated into the Bluescript KS- plasmid and transformed into DH5α competent cells. Individual clones were sequenced at the Cornell University Life Sciences Core Laboratory Center (Ithaca, NY).

### Phylogenetic analysis

We produced a phylogeny of corticosteroid receptors using MrBayes 3.1.2 [48], run at the facilities of the Computational Biology Service Unit at Cornell University. In addition to the sequences used in this study, we added sequences from several other fish species for phylogenetic resolution: *Myxine glutinosa *(hagfish) corticosteroid receptor (GenBank no. DQ382336.1), *Petromyzon marinus *(sea lamprey) corticosteroid receptor (GenBank no. AY028457), *Leucoraja erinacea *(little skate) MR (GenBank no. DQ382339.1), *L. erinacea *GR (GenBank no. DQ382338.1), *Oncorhynchus mykiss *(rainbow trout) MRa (GenBank no. AY495584.1), *O. mykiss *MRb (GenBank no. AY495585.1), *O. mykiss *GR1 (GenBank no. Z54210.1), *O. mykiss *GR2 (GenBank no. AY495372.1), *Astatotilapia burtoni *(Burton's cichlid) MR (GenBank no. AF263741), *A. burtoni *GR1b (previously GR2b; GenBank no. AF263740), and *A. burtoni *GR2 (previously GR1; GenBank no. AF263738.1). An additional GR isoform, GR2a, from *A. burtoni *was not included because it is a alternatively spliced isoform of the same GR1 gene which produces the GR1b transcript used in this analysis [12]. A phylogram was created based on an alignment of LBD and hinge regions as well as full length coding sequences when they were available. A nucleotide alignment, which was used for the phylogenetic analysis, was aligned based on the deduced amino acid sequences using Clustal W. A general time reversible model with invariable sites and a gamma distribution for variable rate sites (GTR+I+G) was used. Four Markov chains of 1,000,000 generations sampling every 100^th ^generation with a burn-in of 25% were used for the analysis. A majority rule consensus tree and posterior probabilities were generated. The resulting phylogram was visualized and edited Mesquite 2.5 [49].

### GAL4-UAS transactivation constructs

The GAL4 DBD, engineered with a FLAG epitope tag (DYKDDDDK) at its amino terminus, was amplified and cloned into the pBluescript KS- plasmid. The corticosteroid receptor LBDs were each subcloned in frame with the GAL4 DBD in pBluescript KS- to produce gene cassettes coding for single FLAG-tagged GAL4 - LBD fusion proteins. Each of these cassettes were then subcloned into the pCMV5 mammalian expression vector under regulation of the constitutive CMV promoter. To produce the luciferase reporter construct, a DNA region isolated from a pUAST vector containing five repeating GAL4 UAS domains with a minimal HSP70 promoter was inserted into a pGL3-luc vector upstream of the luciferase coding region. These constructs were all sequenced to verify their accuracy before maxi-prepping them for use in Western blot and transactivation analyses.

### Western blot analyses

The methods used were similar to those reported by Kim et al. [50]. Briefly, HeLa cells were grown in DME/F-12 (Invitrogen, Carlsbad, CA) containing 10% CDCS. The cells were plated in six-well plates and grown to approximately 70% confluency. Each well was transfected with 400 μg of pCMV5-GAL4-LBD using GeneJuice transfection reagent (Merck KGaA, Darmstadt, Germany). Eighteen h after transfection, the cells were harvested in protein loading buffer (62.5 mM Tris pH 6.8, 1% SDS, 5% glycerol, 65 mM DTT, 0.02% bromophenol blue). Cell extracts were heated to 95°C for 5 min, run out on a 10% polyacrylamide gel, transferred to PVDF membrane, and detected using the FLAG M2 antibody (Sigma-Aldrich, St. Louis, MO).

### Transactivation assays

The methods used were similar to those reported previously [50]. Briefly, HeLa cells were grown in DME/F-12 (Invitrogen, Carlsbad, CA) containing 10% CDCS. The cells were plated in six-well plates and grown to approximately 70% confluency. The cells in each well were co-transfected with 500 ng of the pGL3 GAL4 regulated luciferase reporter construct and 400 ng of one of the pCMV5-GAL4-LBD constructs. An initial screen of corticosteroid receptor ligand sensitivity of the midshipman and mouse receptors was performed 12 to 24 h after transfection by treating the cells with vehicle (ethanol) or 10^-7 ^M of one of several steroid hormones. Cells were incubated for an additional 18 h.

The hormones examined included aldosterone, dexamethasone, hydrocortisone (cortisol), 11-deoxycortisol, cortisone and the non-aromatizable androgen 11-ketotestosterone (11-KT). The 11-deoxycortisol and cortisone were used to examine cortisol specificity because they are the immediate upstream and downstream steroids, respectively, of cortisol in the steroid biosynthetic pathway [1]. Dexamethasone, a synthetic glucocorticoid, was used with the GR constructs because of its well-established high binding affinity for mammalian GR [21,51,52]. However, dexamethasone was not used with MR constructs because the dexamethasone paradox, the finding that MRs have variable affinities for dexamethasone *in vitro *versus *in vivo *[review: [53]], would have confounded the interpretation of results in our heterologous expression system. The androgen 11-KT was chosen to examine whether the isolated LBDs show generalized binding to C3-keto steroids; 11-KT is also a predominant androgen in midshipman [54]. To examine whether mineralocorticoid sensitivity was consistent with the phylogeny of teleost corticosteroid receptors, we also performed these transactivation assays on the daffodil cichlid MR, GR1, and GR2 with aldosterone and cortisol.

In addition to the discriminatory capacity of the LBDs, we examined the relative ligand sensitivities of the corticosteroid receptors of midshipman and mice by incubating transfected cells for 18 h with 10-fold dilutions ranging from 10^-11 ^to 10^-6 ^M of aldosterone, 11-deoxycorticosterone (DOC), dexamethasone, or cortisol. DOC, an aldosterone precursor, was selected because it may be the dominant mineralocorticoid in teleost fish [10]. The dose response analyses were performed on cells co-transfected with the GAL4 regulated luciferase reporter construct and the pCMV5-GAL4-LBD constructs from mouse and midshipman.

After 18 h the cells were washed with PBS and lysed using1x Lysis buffer (Promega, Madison, WI). The luciferase activity of each cell extract was measured using a mix of 50 μl luciferin (1:1 with water) and 50 μl extract. The raw luciferase values for each fusion product were normalized to the values for the vehicle (ethanol) treatment specific for that LBD. To ensure reproducibility, each assay was run in duplicate, and each experiment was performed at least three times.

### Protein sequence and structural analyses

To investigate the molecular basis for variation in mineralocorticoid sensitivity of GRs, we aligned the amino acid sequences of LBDs from GR1 and GR2 of midshipman and daffodil cichlid with GR2 of Burton's cichlid and rainbow trout GR1, two other species with substantial documentation of the ligand sensitivity of corticosteroid receptors [2,10,12,13]. Burton's cichlid GR1 and rainbow trout GR2 were excluded because their mineralocorticoid sensitivities are less clear based on previous studies [2,12,13]. We used this alignment to identify amino acid substitutions that correlated with mineralocorticoid sensitivity across GRs. To more closely examine the potential effects of identified amino acid substitutions on the protein structure and ligand-binding pocket we created homology models of the GR ligand binding domains using the SWISS-MODEL homology-modeling server and DeepViewer 4.0 software [55-58], using as templates the crystal structures of a putative ancestral GR in complex with dexamethasone (PBD ID: 3GN8) as well as fitting an ancestral corticosteroid receptor in complex with aldosterone (PDB ID: 2Q1H) and cortisol (PDB ID: 2Q1V).

### Statistics

One sample t-tests were performed to test whether each normalized luciferase activity was greater than the baseline of 1.0 at 10^-7 ^M treatment with each ligand. We performed our dose response analyses for each LBD construct by fitting three-parameter logistic curves of the form $f(x)=\beta_{1}+\frac{1-\beta_{1}}{1+{\left(x/\beta_{2}\right)}^{\beta_{{}_{3}}}}$ to our data where *f*(*x*) is the response to dose *x*, *β*_1 _is the estimated upper bound on the response, *β*_2 _is the estimated EC_50_, and *β*_3 _is the estimated "slope" or shape parameter. Since our responses were normalized to the LBD specific vehicle treatment, the zero dose response was set to 1 in our model. However, because the midshipman GR2 construct showed significant activity with all the corticosteroids the typical 4-parameter curve was used for this construct because it produced a more accurate fit to the data. Many LBD constructs showed a previously observed decrease in responses at high doses after the initial increase which may result from cytotoxicity at these doses [32]. We accounted for this drop by excluding such points from our analysis; for each construct, we calculated the dosage, *d*_*M*_, at which the maximum average response was achieved and included only those data points with doses at or below *d*_*M *_in our curve fitting algorithms. The fitted curves were obtained using the least squares curve fitting program in MatLab's optimization toolbox (Natick, MA) and the built in non-linear least squares curve fitting program in R [59]. Since fitting nonlinear curves may converge to local minima in the landscape of possible solutions, we tested the robustness of our fits by running our curve fitting programs multiple times with different initial values for the three-parameters *β*_1_, *β*_2_, and *β*_3_. We quantitatively assessed each fit by computing the residual sum of squares (RSS) and selected the model with the smallest RSS. In some cases, two models yielded similar RSS and in these cases, we selected the model which gave the most reasonable qualitative fit. All graphs were produced with SigmaPlot 10 (Systat Software Inc, San Jose, CA).

## Authors' contributions

ASA, DLD, WLK, and AHB conceived of the study. ASA, DJF, EAF, DLD, WLK, and AHB designed the study. ASA, DJF, and EAF carried out molecular and transactivation studies. ASA and DJF performed sequence and structural analyses. JM performed statistical analyses. ASA, DJF, and AHB drafted the manuscript. All authors assisted with data interpretation, and edited and approved the final manuscript.
